# Supplementary figures and images for: Immunogenicity and Protective Capacity of Sugar ABC Transporter Substrate-Binding Protein against Streptococcus suis Serotype 2, 7 and 9 Infection in Mice
Source: Vaccines (Basel). 2024 May 15;12(5):544. doi: 10.3390/vaccines12050544 (PMC11126002; doi:10.3390/vaccines12050544)

A

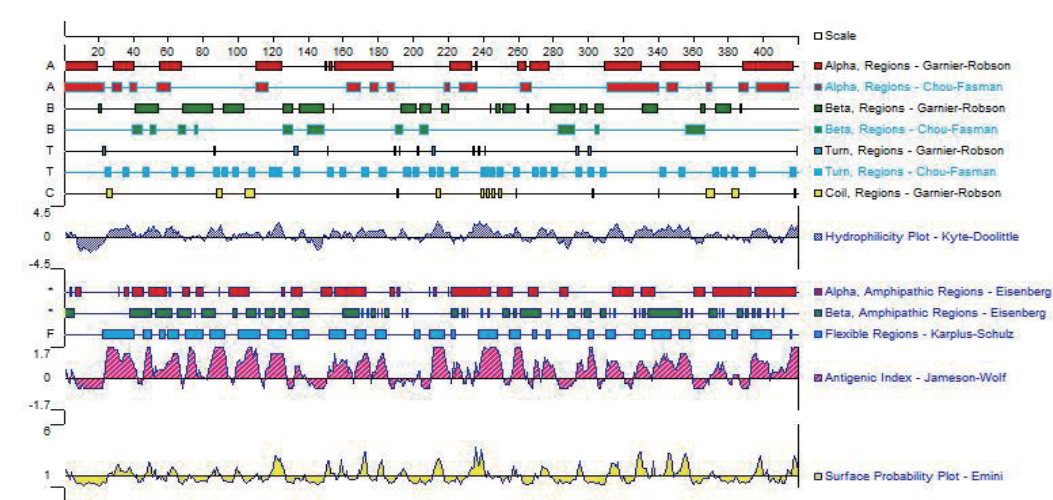

B

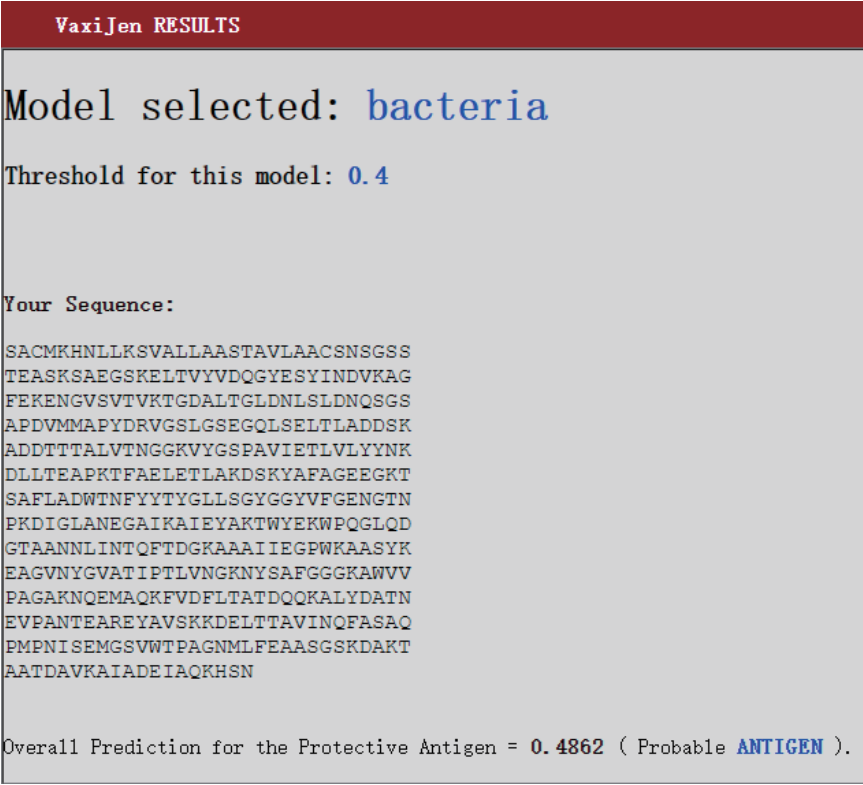

Figure S2. Antigenicity analysis.

Supplement: Supplementary file 1 [file vaccines-12-00544-s001.zip › vaccines-2942452-supplementary/vaccines-2942452 - Supplementary Fig. 2.pdf]

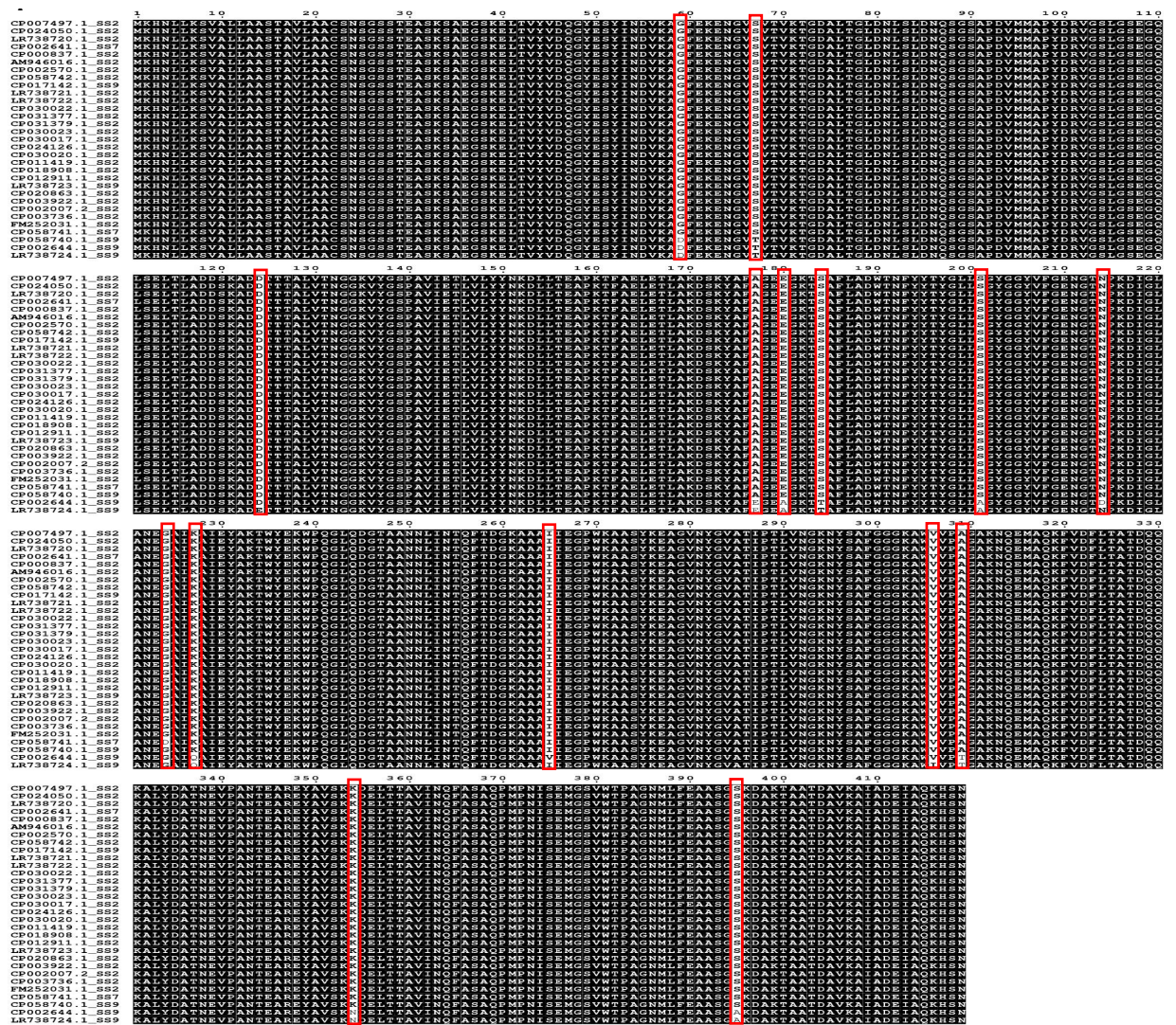

Figure S1. Conservation of S-ABC gene in *S. suis* strains.

Supplement: Supplementary file 1 [file vaccines-12-00544-s001.zip › vaccines-2942452-supplementary/vaccines-2942452 - Supplementary Fig.1.pdf]
